# Inlay Investment*Extract from an article by Dr. Travis, which is printed in full in the December *Journal N. D. A*.

**Published:** 1920-01

**Authors:** J. J. Travis


### INLAY INVESTMENT *

BY J. J. TRAVIS, D.D.S.

There are several good investments on the market, any of which will give good results when we have learned how that particular investment should be manipulated. Dr. Taggart's investment gives you more time, gets harder, makes a more resistant mold and gives finer detail than any other investment I have used. It has the disadvantage, however, of requiring more care to work the air out.

The more porous the investment, the less distinct the detail and the easier it will be to manipulate. Much work has been done on investments and many changes in formula made, but it is universally conceded now that the ideal foundation is a pure form of silica free from ferric oxide.

Silica is used because of its great strength and its high fusing property. A coarse and finer grade are thoroughly mixed and only enough matrix, or cementing material, is used to hold the silica crystals in contact. The higher the percentage of silica, the less the volume changes and the more you fill in the interstices with graphite and plaster, the finer the detail. Fine detail

* Extract from an article by Dr. Travis, which is printed in full in the December *Journal N. D. A.*

means low porosity and calls for a higher pressure to expel the air from the mold. The only cementing element in any investment is plaster of Paris and like any plaster model the thicker it can be mixed, giving you time to manipulate it, the less excess water and the greater crushing test it will give.

Dr. Taggart's inlay investment, if mixed according to direction, gives you eight minutes in which to complete the investment.

A more resistant mold can be made with less disturbance of the crystals in drying and burning out, if the percentage of water is slightly diminished, so I usually take out a few drops of water from the balance before mixing. This, of course, shortens the time but if your assistant will paint the model while you manipulate the investment, better results will be obtained. I do not believe that the plaster bowl should be allowed to sit quietly on the desk while you paint the model, for crystallization proceeds much more rapidly while at rest.

We should endeavor to complete the investment before crystallization begins if we expect to get the strongest possible mold.

### SPATULATION

It has been shown that longer spatulation gets the elements closer together and makes a stronger mold. Dr. Taggart recommends spatulating one minute and jarring and rotating in the bowl for two minutes more to allow bubbles to escape. By increasing the time of spatulation to two and one-half minutes an investment will result that will show greater crushing strength. Two inlay rings were filled with investment, one of which was spatulated one minute and the other two and one-half minutes and allowed to set one hour. After they were burned out in an electric furnace together and tested for hardness with a delicate scleroscope, I found the difference not to be great, but slightly in favor of the one that was spatulated longest. Similar deductions were drawn when tested with the dynamometer.

Investment mixed in this manner should set one hour and is then ready for drying.

To do this intelligently, we should know the nature of the materials with which we are dealing.

### DRYING AND BURNING OUT

All of the ingredients of an investment except plaster are unaffected by any temparature employed in the burning out process, but plaster is weakened by every degree of heat above that necessary to drive out the uncombined or mechanical water.

I am inclined to believe that the term ''mechanical water'' is a delusive one because most men think that all water driven off before the temperature of 800 degrees Fahrenheit (the temperature of disassociation of plaster) is mechanical water.

An elucidating experiment can be performed which I think will clear up this misapprehension by taking a piece of gypsum from which plaster is made and applying heat to it. Moisture, which is the water of crystallization, will begin to escape at about 150 degrees C. and will continue until calcination has progressed to the point where quick setting plaster is formed. The block of gypsum has now changed from a translucent mass to a pure white block.

If this is ground in the mortar, a good grade of plaster results. You will note, however, that the escape of water has been a gradual and continuous process as the temperature rises.

The resulting white powder will take up water again and recrystallize, setting into a hard mass. If you again apply heat the same phenomenon occurs and the water which we now know to be water of crystallization will be gradually driven off as the temperature rises.

Now this can not be done except at the expense of strength and we should be very careful not to subject the investment to any more heat or for any longer period than just enough to drive off the volatile elements from the burning wax. The resulting carbon need not all be burnt out as it will in no way injure the cast, but will act as a deoxidizing agent when casting to other metals and make a smoother cast by filling up the pores of the surface of the mold.

The main thing is to see that the investment is never subjected to temperatures higher than are necessary to burn out the wax to this degree.

A direct flame should never play upon the investment.

Another factor that causes many rough casts is the fact that heat is applied too rapidly and the wax becomes molten before the investment is dry enough to allow the molten wax to soak back into the investment. Molten wax will not penetrate wet investment.

This temperature should be approached slowly for wax will boil in a wet mold and destroy all of the detail. Taggart's wax softens at 70 degrees C. and is entirely liquid at 80 degrees C. At this temperature it will thoroughly permeate the investment with no injury to the surface of the mold.

Rapid rises in temperature are dangerous particularly when large quantities of wax are employed. It should be given time for movement into the investment. The wax begins to boil at 320 degrees C. and we should be sure that it never occurs in the mold cavity. A delicate pyrometer will show that smoking begins at about this temperature and all of the volatile elements can be driven off without increasing the heat beyond this point.

You will readily apprehend from these facts that some method of applying heat other than the Bunsen burner is necessary because it is so difficult to control or measure.

I believe we will be getting better fits and smoother inlays when we adopt some method of heating where the temperature can be perfectly controlled and the heat known.

An electric furnace is ideal for this purpose for the changes are gradual and under perfect control.

I have conducted my simple experiments in the physical laboratory in the dental college where I had an excellent furnace, a rheostat, and a very delicate pyrometer to control and measure the heat. This would be too expensive a piece of apparatus for the average practitioner to have in his office, but with it one can determine what heats are most efficient and the time required.

The most excellent results were obtained when considerable time was taken and the heat increased more rapidly in the later stages of the process. At no time was it necessary to increase the heat above 320 degrees C. or 608 F. At this temperature smoke began to appear and we did not allow the temperature to rise any higher, but maintained it until the volatile elements were driven off or until it had practically stopped smoking.

The time required for this stationary temperature varies with the quantity of wax, from ten to fifteen minutes.

I had four inlay rings split so that I could invest, burn out and separate through the center of the mold, showing the degree of carbonization obtained, at five, eight, ten and fifteen minutes heating after smoking began.

I used the same cavity to carve the wax pattern from in each case. The first showed little discoloration, the second a distinct brown, the third a lighter brown and the fourth a nearly white mold.

Inlays cast when the mold had reached the third stage possessed a smoother surface than the overbaked molds and went to their seating more easily. Excessive heating seems to have increased the porosity of the surface and weakened the investment.

The experiments I have performed have not been of a highly scientific nature, but have rather been the inquiries of a practical operator seeking information on a perplexing problem daily encountered in the dental clinic. There is not at present, I believe, on the market a furnace perfectly adapted to our needs, but a growing demand should soon induce some of our dental supply houses to manufacture one. I am inclined to think that such a furnace should have more than one heat—that is, it should have a simple rheostat attached and some means of knowing when the maximum temperature has been reached with power to control it.

In baking porcelain, the gas flame, because of its variableness, had to be discarded as a heating agent and now electricity has completely superseded it.

Since my experiments with the electric furnace, I have tried to protect the investment from the flame in an improvised oven heated by gas. While I had no means of knowing the temperatures, I kept the heat low and maintained it for about an hour with gratifying results.

We may be certain that we are doing the investment no harm before smoking begins but after that we should watch it closely.

If a bare flame plays upon a cube of gypsum you will note that it begins to turn white on the corners and edges first, gradually progressing inward. The outside crystals become weakened first and this is probably what takes place in the investment. The mold cavity which is nearest the bottom of the ring will be affected by a direct flame before we realize it.

In conclusion, allow me to state that in my opinion electricity is our best agent for burning out the wax, but any agency by which a perfectly controlled heat may be secured with a thermometer or pyrometer attachment, will enable us to make smoother and better-fitting inlays than we have been getting by the usual methods.

